# Dopamine D1 receptor blockade impairs alcohol seeking without reducing dorsal striatal activation to cues of alcohol availability

**DOI:** 10.1002/brb3.305

**Published:** 2015-01-05

**Authors:** Rebecca R Fanelli, Donita L Robinson

**Affiliations:** 1 Neurobiology Curriculum, University of North CarolinaChapel Hill, North Carolina; 2 Bowles Center for Alcohol Studies, University of North CarolinaChapel Hill, North Carolina; 3 Department of Psychiatry, University of North CarolinaChapel Hill, North Carolina

**Keywords:** dopamine D1 receptor antagonist SCH23390, dorsolateral striatum, dorsomedial striatum, extracellular electrophysiology, RRID:nif-0000-31484, RRID:nlx_153890, RRID:nlx_157643, RRID:nlx_158483, RRID:nlx_158484, RRID:nlx_158504

## Abstract

**Introduction:**

Alcohol-associated cues activate both ventral and dorsal striatum in functional brain imaging studies of heavy drinkers. In rodents, alcohol-associated cues induce changes in neuronal firing frequencies and increase dopamine release in ventral striatum, but the impact of alcohol-associated cues on neuronal activity in dorsal striatum is unclear. We previously reported phasic changes in action potential frequency in the dorsomedial and dorsolateral striatum after cues that signaled alcohol availability, prompting approach behavior.

**Methods:**

We investigated the hypothesis that dopamine transmission modulates these phasic firing changes. Rats were trained to self-administer alcohol, and neuronal activity was monitored with extracellular electrophysiology during “anticipatory” cues that signaled the start of the operant session. Sessions were preceded by systemic administration of the D1-type dopamine receptor antagonist SCH23390 (0, 10, and 20 *μ*g/kg).

**Results:**

SCH23390 significantly decreased firing rates during the 60 s prior to cue onset without reducing phasic excitations immediately following the cues. While neuronal activation to cues might be expected to initiate behavioral responses, in this study alcohol seeking was reduced despite the presence of dorsal striatal excitations to alcohol cues.

**Conclusions:**

These data suggest that D1 receptor antagonism reduces basal firing rates in the dorsal striatum and modulates the ability of neuronal activation to “anticipatory” cues to initiate alcohol seeking in rats with an extensive history of alcohol self-administration.

## Introduction

Cues can play a powerful role in addiction, triggering craving, drug seeking, and relapse (Le and Shaham 2002; Volkow et al. 2006; Corbit and Janak 2007). In human functional MRI studies, alcohol cues activate both ventral and dorsal striatum (Filbey et al. 2008). In ventral striatum of rodents, alcohol-associated cues can trigger increases in neuronal firing rates (Janak et al. 1999; Robinson and Carelli 2008) as well as dopamine release (Weiss et al. 1993; Gonzales and Weiss 1998; Howard et al. 2009). Less is known of the neurobiology of dorsal striatal activity in response to alcohol-associated cues. However, the dorsal striatum receives spiraling, feed-forward input from the ventral striatum via midbrain dopamine neurons (Haber et al. 2000), and the dorsal striatum is known to be essential for updating reward value and for action selection (Haber et al. 2000; Yin and Knowlton 2006; Devan et al. 2011).

The dorsal striatum is functionally heterogeneous, with the dorsomedial striatum (DMS, homologous to the primate caudate) required for learning relationships between actions and outcomes and the dorsolateral striatum (DLS, homologous to the primate putamen) necessary for stimulus–response associations and becoming increasingly engaged later in learning (Yin et al. 2005a, 2006; Kimchi et al. 2009; Corbit et al. 2012). These functions also depend on dopamine. Systemic D1 receptor antagonism with SCH23390 blocks the reinforcing effects of cocaine and reduces motivated behavior (Koob et al. 1987; Weissenborn et al. 1996; Liu and Weiss 2002). In the DMS, antagonism of D1 receptors reduces the ability of a reward to modulate behavior (Nakamura and Hikosaka 2006). Additionally, interruption of the dopaminergic inputs to the DLS can prevent habit formation (Faure et al. 2005) and reduce habit-like cocaine seeking (Belin and Everitt 2008). Therefore, we hypothesized that dopamine transmission via D1 receptors in the dorsal striatum may directly modulate excitatory neuronal activation to alcohol-associated cues, while altering alcohol seeking.

To investigate engagement of the dorsal striatum by alcohol cues and during alcohol seeking, we previously performed in vivo extracellular electrophysiology during alcohol self-administration in rats and monitored neuronal firing patterns (Fanelli et al. 2013). We found that the DMS predominantly demonstrated phasic excitations to cues, while the DLS was activated around lever-press responses. Start-of-session cues elicited phasic activation of both DMS and DLS neurons and behavioral approach responses. Since the D1-expressing direct-pathway neurons in the striatum express D1 receptors and contribute to initiation of behavior while D2-expressing indirect-pathway neurons inhibit behavior (Freeze et al. 2013), we expected that D1 receptor antagonism would blunt the observed dorsal striatal activation. The present study tested the effect of the D1-like receptor antagonist SCH23390 (SCH) in rats with continued, stable operant behavior, from which DMS and DLS neuronal activity during typical self-administration training sessions was previously reported. SCH was administered prior to alcohol self-administration sessions during which we used electrophysiology to record neuronal activity in the DMS and DLS. Antagonism of D1-like receptors inhibited alcohol-seeking behavior and reduced basal firing rates without preventing neuronal excitations to alcohol-associated cues, suggesting an uncoupling of phasic neuronal encoding and behavioral responses. As addiction can result in a hypodopaminergic state (Koob 2009; Morikawa and Morrisett 2010), enhanced signal-to-baseline ratios seen here after D1 receptor antagonism may be important for processing and adaptive learning in addiction.

## Methods

### Subjects

Adult male Long-Evans rats (250–300 g) were purchased from Charles River (Raleigh, NC) or Harlan (Indianapolis, IN). Rats were individually housed under a 12 h:12 h light:dark schedule and received food and water ad libitum except for the first 5 days of operant training, when they were water restricted for 23 h/day. All procedures were conducted in accordance with the NIH Guide for the Care and Use of Laboratory Animals and approved by the Institutional Animal Care and Use Committee of the University of North Carolina.

### General alcohol self-administration procedures

Rats underwent sucrose-fading and procedures in order to self-administer 10% w/v ethanol. Rats were trained in one 30-min session each day, Monday - Friday, in custom-built Plexiglas operant chambers in sound-attenuating cabinets (MedAssociates, St. Albans, VT) as previously described (Fanelli et al. 2013). Rats initially entered operant chambers 5 min prior to the session start, and this habituation period was lengthened to 15 min by the time of electrophysiological recordings, to allow the experimenter to set recording parameters and choose a differential reference. Important for this study, sessions began with the illumination of the house light and extension of the levers into the operant chamber 30 s later; these stimuli signaled the start of the operant session and predicted alcohol availability. Reinforcer deliveries of 0.1 mL were paired with a cue light located above the response levers.

Rats were trained to respond on an FR5 (every fifth lever-press response = 1 fluid delivery) or a VI30 schedule (after a variable interval averaging 30 s has elapsed, the first response = 1 fluid delivery). Responses on only one lever (either right or left) triggered fluid delivery and cue light illumination, while the other lever was inactive (responses were recorded but had no programmed consequences). More details can be found in our previous report (Fanelli et al. 2013). Sessions ended after 25 reinforcements were earned or after 30 min, whichever came first.

### Surgery

After at least 6 weeks of training, rats that maintained stable self-administration behavior were implanted with 16 stainless-steel, Teflon-coated electrodes (50 *μ*m diameter, 500 *μ*m apart; NB Labs, Denison, TX) as previously described (Fanelli et al. 2013). Oriented anterior to posterior with electrodes linearly aligned, electrode arrays targeted the DMS (+0.2 to +2.0 mm AP, ±1.7 mm ML, −4.5 mm DV from bregma), and the contralateral DLS (+0.2 to +2.0 mm AP, ±3.4 mm ML, −4.5 mm DV), with sides counterbalanced across rats. After surgery, rats were given 15 mg/kg ibuprofen daily for 3 days and allowed a week to recover.

### Electrophysiology

Next, rats were habituated to the tether connecting the electrode arrays to the headstage assembly in operant chambers identical to the training chambers except that they were equipped for electrophysiological recordings. Recordings were analyzed from sessions acquired after operant behavior stabilized. Neuronal activity was recorded using a multichannel acquisition processor (MAP system; Plexon, Inc., Dallas, TX). Neural activity was recorded simultaneously from the 16 electrodes (RASPUTIN, Version 2.2.0, Plexon, Inc. RRID:nlx_158504). Briefly, a differential reference electrode was designated on each array. Cell sorting was finalized after the experiment with Offline Sorter software (Offline Sorter, Version 2.8.8, Plexon, Inc. RRID:nlx_158484; further described in Fanelli et al. 2013). Automated clustering based on template analyses and principle component analysis (PCA) was manually adjusted, guided by observations made during data collection (Robinson and Carelli 2008; Fanelli et al. 2013). Signal-to-noise ratios ≥2 (online), distinct PCA clusters (offline), and physiological characteristics consistent with medium spiny neurons (i.e., ≤0.1% of spikes with interspike intervals <1 ms and average firing rates <10 Hz; Kimchi et al. 2009; Kish et al. 1999) were required for inclusion of neurons in analyses.

### SCH23390 effects on self-administration and neuronal activity

After initial electrophysiological recording of the baseline operant session as previously reported (Fanelli et al. 2013), electrophysiological data were recorded during operant sessions after administration of SCH23390 (Sigma-Aldrich, St. Louis, MO) or vehicle. Only rats that maintained stable lever-press behavior through the baseline recording session were included in this study. Inclusion criteria required that rats receive >65% of the 25 available reinforcements prior to SCH testing (excluded after receiving <65% for three consecutive sessions). SCH was dissolved in saline vehicle to achieve concentrations of 0, 10, or 20 *μ*g/kg in a final injection volume of 0.3–0.6 mL. Doses were selected that were reported to reduce behavioral responses to cues associated with cocaine and not food-associated cues (Weissenborn et al. 1996). Rats received SCH doses (i.p.) 30 min prior to start of session in a counterbalanced order, with a habituation injection of saline (0.9%) administered on a day prior to the first test. Specifically, as early experiments found that 20 *μ*g/kg often affected operant behavior on subsequent days, the majority of rats received saline and 10 *μ*g/kg SCH in randomized order, followed by the 20 *μ*g/kg dose. SCH test sessions were separated by at least two regular operant sessions.

### Histology

Rats were anesthetized with ≥1.5 g/kg of urethane (50% w/w in saline) before 10 *μ*A current was applied for 5 s to each wire. Rats were perfused, and brains sectioned and stained as previously described to confirm electrode placement (Robinson and Carelli 2008).

### Data analysis

Perievent histograms of firing rates were created using NeuroExplorer (NeuroExplorer, Version 4.088, Plexon, Inc., RRID:nlx_158483), and population analyses were completed using custom-written programs in MATLAB (Version R2008b, The MathWorks, Inc., Natick, MA, RRID:nlx_153890). The average firing rates of all neurons in each region were aligned to each event and smoothed with a moving average of 250 ms in 50 ms steps. Normalized firing rates were calculated through division of each bin by the mean whole-session firing rate. Firing rates around start-of-session events are averaged for each cell, then within each region, and presented as mean ± SE.

The average number of spikes in a target window—the 0.5 s after an event (signal) — was compared to a baseline calculated from the 60 s prior to the start of the session (i.e., prior to houselight illumination). The two start-of-session cues were expected to have similar effects on neuronal activity, and this was confirmed by Wilcoxon Signed Rank Test (Table S1); consequently, light, and lever presentations were treated as two observations of the same event (cue signal). Neuronal activity from FR5- and VI30-trained rats were compared for firing rate (raw, non-normalized) and coefficient of variance in the baseline, as well as firing rates in the signals (averaged and individually) with 2-way ANOVA, and main effects were examined with Holm–Sidak post hoc multiple comparison method. These analyses yielded no significant effects of group (Table S2); consequently, the groups were combined for subsequent neuronal activity analyses. The effect of SCH dose on signal and baseline in DMS and DLS was tested by parametric multivariate regression analysis [GENMOD procedure, with a Poisson-distribution regression model with repeated measures (RM) and using a log transform of time to account for differences in the time window for signal vs. baseline]. Main effects, interactions, and pairwise contrasts were compared with the Wald test (Dorsal striatal activation to cues SAS 9.3, SAS Institute, Inc., Cary, NC, RRID:nif-0000-31484). Cell detection rates in each rat and brain region were compared by 2-way RM ANOVA. The proportion of individual neurons showing altered firing rates around events was calculated using z-scores comparing phasic frequency in the 0.5 s after the cues to the prior 60 s baseline. A significant change in firing rate occurred when |*Z| *≥ 2.

Operant behavioral data are presented as mean ± SE. Latency to the first press, lever press responses, and reinforcements earned were compared across sessions with the Friedman ANOVA on ranks with repeated measures (Sigma Plot, SyStat Software, Inc., San Jose, CA, RRID:nlx_157643). Post hoc contrasts were made with the Tukey test for multiple comparisons. Spearman rank order correlation examined the relationship between behavioral measures (latency to the first press, lever presses, and EtOH deliveries earned) and the number of action potentials during the signal and the baseline epochs (Sigma Plot).

## Results

**Figure 1:**
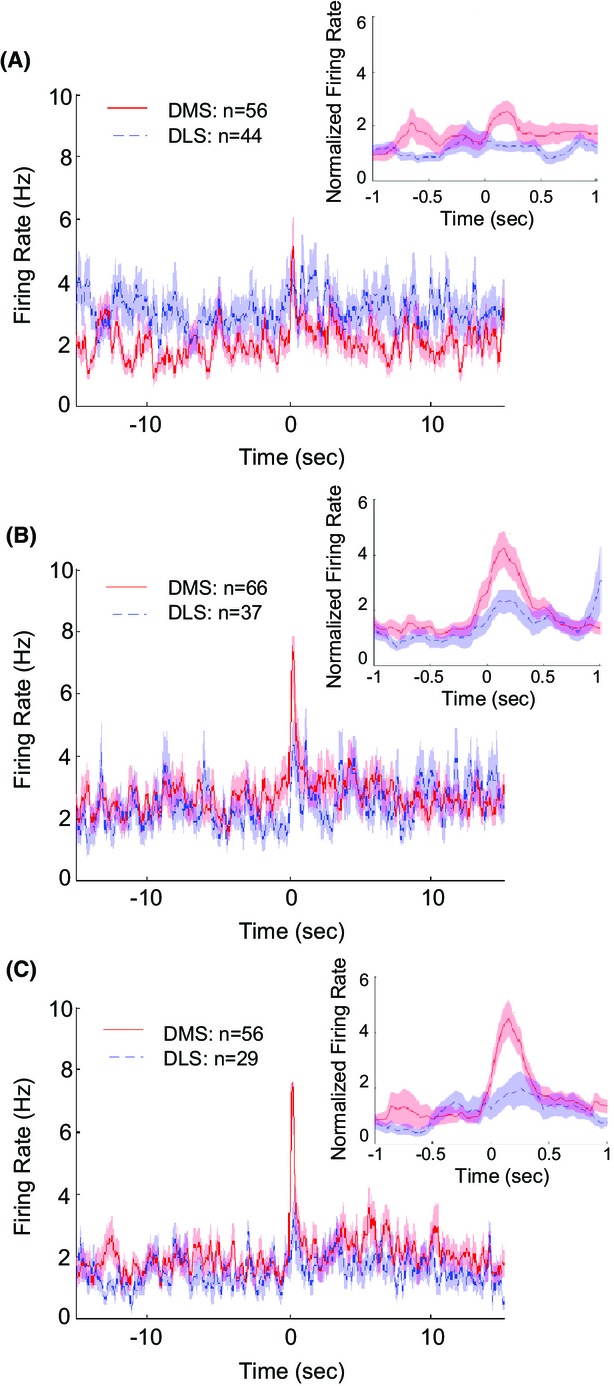
Neuronal firing rates (Hz) in the DMS (red, solid line) and DLS (blue, dotted line) averaged across the two cues and aligned to start-of-session cues (at 0 s). Mean firing rates (±SEM shaded) recorded during self-administration sessions following (A) saline, (B) 10 *μ*g/kg SCH, and (C) 20 *μ*g/kg SCH. Insets display a 2 s window to focus on cues (at 0 s), with the firing rate of each neuron normalized to the whole-session firing rate.

In order to investigate the contribution of D1 receptor activation to dorsal striatal neuronal firing in response to alcohol-associated cues, we administered 0, 10, and 20 *μ*g/kg SCH i.p. to 26 rats in a within-subject design (one rat ceased tolerating the tether and did not undergo the 20 *μ*g/kg dose). For this and the previous study, 24 FR5 rats underwent surgery and 14 completed baseline recordings (Fanelli et al. 2013). 11 rats met subsequent performance criterion and were included in this study. For the VI30 group, 21 rats underwent surgery, 16 completed baseline recordings (Fanelli et al. 2013), and 15 met performance criterion and were included in this study. We first tested whether training schedule affected firing rates at baseline and at stimulus presentations. As shown in Table S2, there was no effect of group on these firing rates, so data were combined across training groups. We next determined the effects of SCH on the number of putative medium spiny neurons detected and their basal firing rates. While fewer cells were detected per electrode wire in the DLS than the DMS (main effect of region: *F*_1,24_ = 8.64, *P *<* *0.01), SCH did not significantly alter the number of neurons detected (main effect of dose: *F*_2,24_ = 0.93, *P *=* *0.4, and dose by region interaction: *F*_2,24_ = 0.53, *P *=* *0.6). The number of cells recorded per rat on a given day ranged from 1 to 9 neurons; see Fig.1 for total cell numbers after each dose.

As the primary goal of this study, we analyzed phasic firing changes to stimuli associated with the operant session that were not contingent on the animals' behavior. Specifically, all alcohol self-administration sessions began with the illumination of the house light followed 30 s later by the extension of the operant levers into the chamber, providing cues of alcohol availability that were independent of behavioral activity. Average neuronal firing rates around the presentation of these two cues (averaged across cue type) in the DMS and DLS across SCH doses are shown in Fig.1 (a smaller time window is displayed in the insets, plotted as normalized firing rates). Firing rates increased at cue presentation (at time 0) compared to the frequency before and after, and these cue-related increases were larger in the DMS. Increases after cue presentation appear larger after SCH treatment, and baseline appears lower after both doses, particularly in the DLS.

**Figure 2:**
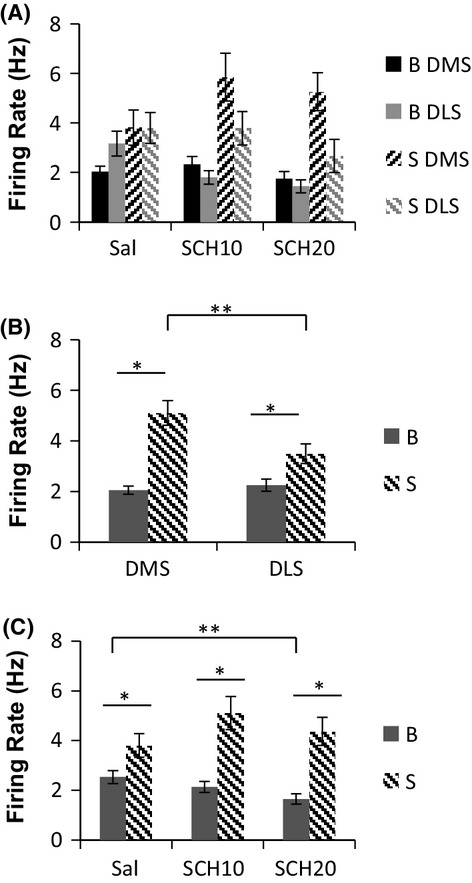
Neuronal firing rates during baseline in the 60 s prior to cue onset (B, solid bars) and the mean signal in the 0.5 s after each of the two cue onsets (S, hashed bars) are displayed (A) for the DMS (black) and DLS (gray) and grouped by dose: saline (Sal), 10 *μ*g/kg SCH (SCH10), and 20 *μ*g/kg SCH (SCH20). (B) Collapsed across dose, signal firing rates were greater than baseline (*P *<* *0.0001*), and the DMS signal was greater than the DLS signal (*P *<* *0.05**). (C) Collapsed across region, signal firing rates were significantly greater than baseline (*P *<* *0.001*), and baseline firing rates were significantly reduced under SCH20 (*P *<* *0.005**).

For statistical analysis, we compared firing frequency of DLS and DMS neurons during the 60 s baseline period (B, immediately before the start of session) to the signal firing frequency (S, the 0.5 s after each of the two cue onsets, entered as two observations of the same variable), as shown in Fig.2A. The GENMOD model of firing rates by time, brain region, and dose yielded significant interactions of time by region (*χ*^2^ = 9.59, *P *<* *0.005), and time by dose (*χ*^2^ = 15.39, *P *<* *0.001), with no significant interaction of dose by region or 3-way interaction. To follow up on the time by region interaction, we collapsed across dose and compared firing rates in DMS and DLS in the signal and baseline. Firing rates were greater in the signal than in the baseline when collapsed across dose (main effect of time, *χ*^2^ = 171.37, *P *<* *0.0001, see * in Fig.2B), and the signal was greater in the DMS than the DLS (*χ*^2^ = 5.81, *P *<* *0.05, see ** in Fig.2B). To follow up on the time by dose interaction, we collapsed across region and compared firing rates during baseline and signal by SCH dose. Signal firing was significantly higher than baseline in all conditions (*P *<* *0.001, see * in Fig.2C). SCH treatment reduced basal firing activity, with a significant reduction in firing rate at 20 *μ*g/kg SCH (post hoc Sal vs. SCH20, *χ*^2^ = 9.31, *P *<* *0.005 see ** in Fig.2C). In contrast, SCH did not alter phasic excitations, as the firing rate during the signal was similar in all drug conditions (*P *>* *0.1).

To assess the effect of SCH on the proportion of neurons with phasically altered firing rates after start-of-session cues, we averaged responses to the two cues, again treating them as trials, and categorized each neuron's phasic activity by evaluating changes in firing with z-score statistics. The proportion of cells with significantly increased firing rates after the cues almost tripled after either SCH dose versus Sal (Table 1). Together, these data indicate that D1 receptor blockade generally reduced firing frequency during baseline, but not at cue onset, and thereby increased the relative excitation to noncontingent, predictive cues.

**Table 1:** Percent of individual neurons with significantly altered firing rates after cue presentations

SCH dose, *μ*g/kg	DMS	DLS
0	11% (6/56)	5% (2/44)
10	30% (20/66)	14% (5/37)
20	32% (18/56)	14% (4/29)

Percent of total units in each region with |*Z| *≥ 2 for z-score comparison of the firing rate in the 0.5 s after the cues (averaged responses to light and lever cues) to the 60 baseline prior to the first cue. In parentheses, number of significant neurons over total neurons recorded. Note that all neurons with significant changes in firing rate were excited, rather than inhibited.

**Figure 3:**
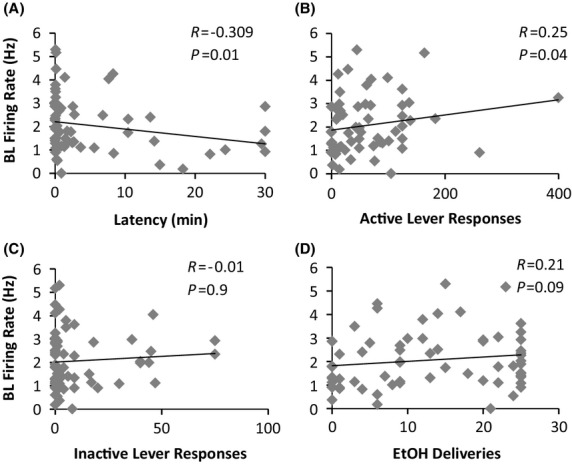
Baseline firing rates (BL, averaged per rat) significantly correlated with (A) latency to the first lever press and (B) the number of active lever responses during the alcohol self-administration sessions (*P*s < 0.05). Therefore, alcohol seeking was slower to initiate and reduced in operant responses in rats with lower firing rates in the 60 s prior to session start. (C, D) Baseline firing rates did not correlate with inactive lever responses or with EtOH deliveries earned during a session.

After presentation of the predictive cues, the D1 receptor antagonist significantly lengthened the latency to the first press (Table 2; *χ*^2^ = 15.6, *P *<* *0.001). Both SCH doses produced significantly longer latency compared to vehicle in post hoc contrasts (all *P*s < 0.05). Furthermore, there was a significant negative correlation between the number of action potentials in the 60 s BL and the latency to the first press (Spearman rank order correlation, *R *=* *−0.309, *P *<* *0.05; Fig.3A); that is, the lower the basal firing rate of the dorsal striatal neurons, the longer the latency for a rat's initial lever press. No such relationship was found between the press latency and S, the neuronal activity after the start-of-session cues (*R *=* *−0.164, *P *>* *0.1).

**Table 2:** Behavioral measures from alcohol self-administration sessions after systemic SCH23390

SCH dose, *μ*g/kg	Latency (s)	Active responses	EtOH deliveries	Inactive responses
0	36.0 ± 20.2	120 ± 15	22 ± 1	21 ± 6
10	297.9 ± 101.0^a^	31 ± 5^a^	9 ± 1^a^	6 ± 2^a^
20	566.5 ± 147.8^a^	15 ± 4^a^	5 ± 1^a^	6 ± 3

Latency to the first press (s), active lever responses, EtOH deliveries earned, and inactive lever responses during alcohol self-administration sessions 30 min after administration of 0, 10, or 20 *μ*g/kg SCH.

a *P* < 0.05 versus 0 *μ*g/kg dose.

Active lever responding was significantly reduced by SCH (Table 2; *χ*^2^ = 38.2, *P *<* *0.001). Post hoc comparisons found that lever presses at each SCH dose significantly differed from vehicle (all *P*s < 0.05), with 75 and 88% reductions after 10 and 20 *μ*g/kg SCH, respectively. SCH also significantly reduced reinforcements earned (Table 2; *χ*^2^ = 41.57, *P *<* *0.001), and post hoc contrasts found that reinforcements after SCH10 and SCH20 were significantly lower than after saline (all *P*s < 0.05). Finally, inactive lever responses were also attenuated by SCH (*χ*^2^ = 8.9, *P *<* *0.05), with inactive lever presses after SCH10 significantly fewer than after saline (*P *<* *0.05), though greater variability at SCH20 prevented a significant reduction from being detected at this dose (Table 2). These behavioral responses were also similar between rats trained on FR5 and VI30 schedules (Table S3). However, because the VI schedule results in a well-established reduction in the rate of reinforcements at baseline (Dickinson 1985; Fanelli et al. 2013), the effect of SCH on the number of reinforcements earned was less significant in this group (Table S3).

Active lever responding also significantly correlated with basal firing rates in the 60 s prior to session start (*R *=* *0.25, *P *=* *0.04; Fig.3B). As with latency, no correlation was found between active presses and neuronal firing rates after the cue signals (S; *P*s > 0.1). Inactive lever responding did not correlate with basal or signal neuronal activity, nor did the number of EtOH deliveries earned (*P*s > 0.05; Fig.3C-D).

## Discussion

We report here that systemic treatment with SCH23390 reduces both basal firing rates of DMS and DLS neurons and alcohol-seeking behavior without attenuating neuronal activation to alcohol-associated cues in rats with extensive alcohol self-administration experience. This treatment reduced inactive responding in a manner consistent with a reduction in effort, as hypothesized. However, we predicted that SCH and interruption of dopamine D1 transmission would reduce neuronal excitations to cues, but an increase in the proportion of significantly excited cells was observed. These findings suggest that dopamine modulates dorsal striatal neuronal activity by altering signal-to-baseline ratio, but is not necessary for neuronal excitations to well-learned cues. Furthermore, as observed in the correlation between the reduction in firing and lever-press number and latency after D1-like receptor antagonism, blocking dopamine transmission may disrupt the link between cue recognition and the initiation of drug-seeking behavior.

Dopamine receptor blockade, systemically or in the ventral striatum, has long been known to suppress ethanol self-administration (Dyr et al. 1993; Hodge et al. 1997). Reward-predictive cues generate dopamine release in the ventral striatum (Roitman et al. 2004; Day et al. 2007; Howard et al. 2009), suggesting that the actions of cues on craving and self-administration (Le and Shaham 2002; Volkow et al. 2006; Corbit and Janak 2007) may also depend on dopamine. For example, tonic dopamine measured by microdialysis increases after rats trained to self-administer alcohol are transferred to the operant chamber prior to ethanol availability (Weiss et al. 1993; Howard et al. 2009). Furthermore, phasic dopamine release in the ventral striatum has been measured proximal to neurons that showed phasic changes in firing rates (Cheer et al. 2005; Cacciapaglia et al. 2011; Belle et al. 2013). Therefore, we hypothesized that dopamine release in the dorsal striatum may provide a mechanism for neuronal activation to reward-associated cues.

Since dopamine release to reward-associated cues has been demonstrated to initiate reward seeking (Steinberg et al. 2013), we expected that dopamine receptor blockade would increase latency to lever-press for alcohol and reduce alcohol seeking. Reward-associated cues are known to initiate reward-seeking behavior (Berridge and Robinson 2003; Cardinal and Everitt 2004; Flagel et al. 2009), and latency to behavioral response has been studied as a measure of behavioral motivation (Wise and Raptis 1985; Blackburn et al. 1987; Liu and Weiss 2002; Salamone and Correa 2002; Morita et al. 2013). Given the involvement of the dorsal striatum in action selection and reward seeking (Haber et al. 2000; Yin and Knowlton 2006; Balleine et al. 2007; Devan et al. 2011), and previously reported correlations between dorsal striatal activation and behavior (West et al. 1990; Kawagoe et al. 1998; Hassani et al. 2001; Fanelli et al. 2013), we expected that neuronal responses to predictive cues in the dorsal striatum would be related to response latency and lever responding. Therefore, we analyzed neuronal responses to cues signaling the initiation of the operant session: houselight illumination and lever extension. These cues evoked similar responses in the dorsal striatum, as previously reported (Fanelli et al. 2013). We found that SCH reduced basal firing rates and not cue-evoked excitations. Moreover, lever-press latency was longer and active lever presses were reduced after SCH administration, and these measures were significantly correlated with basal, but not cue-evoked, firing rates in the dorsal striatum.

Since D1-expressing direct-pathway neurons initiate behavior (Freeze et al. 2013) and increases in striatal firing rates caused by stimulation of dopamine neurons is inhibited by the D1-like receptor antagonist SCH (Gonon 1997), we treated rats with SCH prior to alcohol self-administration sessions. However, while SCH reduced basal neuronal firing, it did not prevent phasic activity to alcohol-associated cues. These data agree with prior studies demonstrating reductions in basal firing rates by SCH (Cheer et al. 2005; Burkhardt et al. 2009), and extend to the dorsal striatum the finding that SCH delivered into the nucleus accumbens increases signal to baseline of phasic excitations through a reduction in baseline (Cheer et al. 2005). Why, then, is phasic dopamine release colocalized with phasically active medium spiny neurons (Cheer et al. 2005; Cacciapaglia et al. 2011; Belle et al. 2013)? One likely explanation is that phasic excitations of firing, such as these activations to well-learned alcohol-associated cues, are facilitated by dopamine release but are primarily glutamatergic. Moreover, the relatively slow time scale of dopamine's actions would promote plasticity and synaptic potentiation of fast glutamatergic synapses (Mangiavacchi and Wolf 2004; see review: Surmeier et al. 2011), rather than an instantaneous modulation of phasic firing. Future studies are necessary to determine whether extended dopaminergic blockade and reduction in basal firing rates would eventually result in reduced neuronal activation to cues, or whether it would prevent further plasticity under conditions requiring behavioral adaptation, such as changes in contingency or reward value. Importantly, the results described here demonstrate that phasic changes in neuronal firing rate are not necessary for changes in subsequent reward-seeking behavior; thus, dopamine may modulate behavior, as by maintaining baseline firing rates, independent of glutamatergic input to alcohol-predictive cues.

Nevertheless, there are a few caveats that are important to discuss. The use of a systemic antagonist treatment raises the possibility that effects were not specific to the dorsal striatum. For example, generalized dopamine blockade may cause nonspecific motor impairment (1 mg/kg; Gimenez-Llort), although 10 *μ*g/kg SCH was previously shown to reduce behavioral responses to cocaine-associated but not food-associated cues (Weissenborn et al. 1996). Herein, as inactive lever presses were reduced by SCH, there may have been a general motor effect impairing operant responding. SCH may also effect dopaminergic neurons in the midbrain, as it has been shown to increase dopaminergic output of the substantia nigra pars compacta (Carlson et al. 1986; Radnikow and Misgeld 1998), where dopamine release would have an amplified effect on D2 receptors (given D1 receptor blockade). D2 receptor activation would, therefore, increase autoreceptor function in addition to activation of the inhibitory indirect pathway, generally reducing movement. SCH thus may indeed reduce dopamine contributions to the dorsal striatum as well as more generally throughout the brain (Belle et al. 2013; Glovaci et al. 2014), and compromised dopamine transmission is associated with deficits in initiating voluntary motor behavior without an external stimulus (Jahanshahi 1998; Choi et al. 2005). Another possible extrastriatal mechanism is the antagonism of D1 receptors in the PFC that may reduce top-down inhibition of striatal cue activation (reviewed in Feil et al. 2010), thereby maintaining phasic, excitatory input to cues that no longer produce an effective behavioral response. Indeed, this may explain the increase in cue-induced neuronal activation after SCH. Supporting this mechanism, muscimol inactivation of the mPFC can increase cue-induced excitations in firing rates in the VTA (Jo et al. 2013). Additionally, SCH effects may occur through other receptors, such as D5 receptors (Bourne 2001) that can colocalize with GABA receptors (another possible mechanism of increased cue responses seen here; Liu et al. 2000). SCH is also a 5HT2 and 5HT1C receptor agonist, though with 10-fold lower affinity (Bourne 2001). Future studies utilizing optogenetic approaches can elucidate the specific role of striatal D1 direct pathways in dorsal striatal encoding of alcohol-associated cues. Thus, while the specific role of dorsal striatal D1 receptors is unclear, we find it interesting that this manipulation was not sufficient to blunt cue responses.

The specificity of SCH to affect the direct pathway may account for the reduction in alcohol-seeking behavior observed here, which is not reflected in the behavior of individuals with alcohol use disorder who may be in a hypodopaminergic state (Koob 2009; Morikawa and Morrisett 2010). Indeed, systemic D1 antagonism can increase tonic DA levels as measured by microdialysis in the DMS (Kurata and Shibata 1991), presumably resulting in enhanced D2 receptor activation. The direct (D1) and indirect (D2) pathways act in parallel, with neurons of each pathway firing in synchrony, such that D1-expressing neurons activate specific action pathways, while D2-expressing neurons deactivate competing pathways (Gremel and Costa 2013; Isomura et al. 2013). Thus, it is possible that the neuronal excitations to alcohol cues observed here may emanate from D2-expressing neurons. However, this would not explain the increase in the proportion of responsive neurons, since we would also expect D2-expressing indirect-pathway neurons to have been active at baseline. Nevertheless, the reduction in alcohol-seeking behavior shown here may result from tipping the scales between the D1/D2 pathways, as blocking only the D1 pathway would result in predominance of the D2 inhibitory pathway. Future investigations will manipulate D2 receptor activation, as antagonism of D2 receptors may reduce alcohol seeking (Weissenborn et al. 1996; Corbit et al. 2014) while exerting bidirectional effects on neuronal activity in the dorsal striatum, since pre- and postsynaptic D2 receptors differ in function (Seeman and Van Tol 1994; De Mei et al. 2009; Beaulieu and Gainetdinov 2011; Anzalone et al. 2012). We expect that higher doses of D2 antagonist, which might target less efficient postsynaptic D2 receptors, would not affect dorsal striatal response to cues, replicating the effects observed in this study. Meanwhile, lower doses of D2 antagonist may have a greater impact on high-efficiency presynaptic receptors, resulting in increases in dopamine neuronal activity and increases in neuronal activation to cues in the dorsal striatum.

Activation to alcohol-associated cues was found in both medial and lateral regions of the dorsal striatum. In a previous experiment, we observed increased population activity in both DMS and DLS to noncontingent, start-of-session cues (Fanelli et al. 2013), and that finding is replicated here. These phasic activations were significantly larger in the DMS, where prior studies have identified neuronal activity related to associative processing (White and Rebec 1993; Rolls 1994). While similar neuronal activation might have been evoked by any novel stimulus, previous reports found that neuronal excitation to a reward-predictive stimulus is amplified to be detectable at the population level only after extended training (Kimchi et al. 2009), whereas habituation would be expected to the repeated presentation of neutral stimuli. Future studies can examine the development of cue-evoked excitations during acquisition and maintenance of operant self-administration. DMS activity observed here may, therefore, reflect encoding of the association of these cues with the initiation of the alcohol self-administration sessions, consistent with the role of the DMS in flexible, goal-directed behaviors (Yin et al. 2005a,b).

Previous studies have demonstrated that DLS activation is related to motor behavior (West et al. 1990) and the DLS is required for habitual behavior (Yin et al. 2006), defined as actions driven by stimulus–response associations (Belin et al. 2009; Devan et al. 2011). Therefore, DLS activation seen here may reflect the ability of anticipatory cues to initiate habit-like approach behavior. While the inhibition of operant behavior was too profound to examine other motor responses here, studies are underway to examine the effect of local dopamine antagonists delivered into the DLS, unilaterally, and bilaterally, on dorsal striatal activity around explicit motor responses such as unreinforced compared to reinforced VI30 lever press responses.

In conclusion, the finding that systemic dopamine D1 receptor antagonism reduced alcohol seeking without affecting phasic cue-related activity has implications for studies of addiction and motivated behaviors. While the electrophysiological data demonstrate that dopamine is not acutely necessary for neuronal activation to conditioned stimuli, the behavioral data suggest that dopamine is important in linking these responses to behavioral activation. Studies of clinical populations with addiction disorders have shown that striatal reactivity to alcohol cues correlates with addiction severity (Filbey et al. 2008), and the reduction in D2 receptor availability in the dorsal striatum in response to cocaine-associated cues correlated with self-reported craving (Volkow et al. 2006). The results of this study suggest that activation of the D1-expressing direct pathway may be responsible for cue-induced drug seeking.

## Supporting Information

**Table S1.**Comparison of neuronal activation to the two start-of-session cue types. **Table S2.** Comparison of the effect of SCH on neuronal firing rates in rats trained on FR5 and VI30 schedules. **Table S3.** Effect of SCH on alcohol-seeking behavior in rats trained to self-administer alcohol on FR5 and VI30 schedules.
