# Supplementary material for: Dopamine D1 receptor blockade impairs alcohol seeking without reducing dorsal striatal activation to cues of alcohol availability
Source: Brain Behav. 2015 Jan 5;5(2):e00305. doi: 10.1002/brb3.305 (PMC4309894; doi:10.1002/brb3.305)
Supplement: Supplementary file 1 [file brb30005-e00305-sd1.docx]

**Supplemental table 1: Comparison of neuronal activation to the two start-of-session cue types.** At the start of operant sessions, the houselight was illuminated followed 30s later by extension of levers into the chamber. We compared neuronal firing upon presentation of each cue to determine whether the neural response was fundamentally different between the two cues or whether the response was similar and the cues could be combined in the statistical analysis. Firing rates in the 500ms after each cue presentation are displayed in Hz (mean ± SEM) and recorded after saline, 10µg/kg SCH and 20µg/kg SCH injections (i.p.) in rats with extended alcohol self-administration experience on FR5 or VI30 schedules. The effects of these session-start cues on neuronal activity were compared by Signed Rank Test within each group (repeated measure, as activity to both cues was recorded from each unit). Overall, we observed similar neuronal activation after light and lever cues (in 10 of 12 comparisons below). Different neuronal responses to the cues were observed in only two conditions: in DMS of VI30 rats under 10 µg/kg SCH, and in DLS of FR5 rats under 20 µg/kg SCH. Thus, as there was no general trend for either cue to be more effective within a region, we treated the two stimuli as repeated trials of the same event (i.e., cue) in our GENMOD analysis to increase the robustness of the analysis.

| **DMS** | FR5 | | VI30 | | |
| --- | --- | --- | --- | --- | --- |
| SCH dose, µg/kg | Light cue | Lever cue | Light cue | Lever cue | |
| 0 | 4.5±1.9 | 3.0±2.4 | 3.4±1.2 | 4.2±1.4 | |
| 10 | 6.6±1.4 | 8.4±1.8 | 3.6±1.2 | 5.6±1.5^a^ | |
| 20 | 5.4±1.7 | 5.4±2.1 | 4.7±1.2 | 5.6±1.5 | |
|  |  | | | |  |
| **DLS** | FR5 | | VI30 | | |
| SCH dose, µg/kg | Light cue | Lever cue | Light cue | Lever cue | |
| 0 | 2.4±1.1 | 4.2±1.3 | 3.9±0.8 | 4.2±1.0 | |
| 10 | 4.3±1.1 | 5.7±1.4 | 2.4±0.9 | 3.7±1.1 | |
| 20 | 0.8±1.2 | 4.3±1.5^b^ | 2.8±1.0 | 2.7±1.2 | |

^a^*W*=293.0, *P*<0.01

^b^*W*=35.0, *P*<0.05

**Supplemental table 2: Comparison of the effect of SCH on neuronal firing rates in rats trained on FR5 and VI30 schedules.** In this study, rats were trained to self-administer alcohol on FR5 and VI30 schedules which are known to generate different operant behavioral strategies (e.g., rates of responding). The dependent measure was neural activity at baseline and upon presentation of the start-of-session cues that were presented independently of behavior. Nevertheless, we compared neural activity between FR5- and VI30-trained rats to determine whether it was fundamentally different between the two groups or whether neuronal activity was similar and the groups could be combined in the statistical analysis. Baseline firing rate (Hz; mean ± SEM) in the 60s prior to the start of the session is presented after saline, 10µg/kg SCH and 20µg/kg SCH injections (i.p.). Neuronal firing frequency was recorded from rats trained to self-administer alcohol on FR5 or VI30 schedules. Separate 2-way ANOVA were run on baseline firing rates, coefficients of variance, and signal firing rates in DMS and DLS to compare group and dose effects. No effects of training schedule were observed in any of the analyses. Specifically, no differences were found in the basal firing rates (30s before presentation of the first cue), in the firing-rate variability during the baseline period (measured with coefficient of variance), nor in the firing rate in the 500ms after cue presentation (average of both cue types). Only significant effects of SCH treatment were observed on baseline firing measures (*P*<0.05).

| **DMS** |  | | | |  | | | |  |
| --- | --- | --- | --- | --- | --- | --- | --- | --- | --- |
| SCH dose,  µg/kg | Baseline Firing Rate | | Coefficient of Variance | | Signal Firing Rate | | |  |  |
|  | FR5 | VI30 | FR5 | VI30 | FR5 | | VI30 |  |  |
| 0 | 2.1±0.6 | 2.0±0.3 | 7.9±1.0 | 9.2±0.6 | 3.8±1.7 | | 3.8±1.0 |  |  |
| 10 | 2.9±0.4 | 2.1±0.3 | 8.9±0.8 | 7.2±0.6 | 7.5±1.2 | | 4.7±1.0 |  |  |
| 20 | 1.4±0.5 | 2.0±0.4 | 7.3±0.9 | 7.2±0.7 | 5.5±1.6 | | 5.2±1.1 |  |  |
| **DLS** |  | | | | |  | | | |
| SCH dose,  µg/kg | Baseline Firing Rate | | Coefficient of Variance | | Signal Firing Rate | | |  |  |
|  | FR5 | VI30 | FR5 | VI30 | FR5 | | VI30 |  |  |
| 0 | 2.9±0.6 | 3.2±0.4 | 7.1±0.8 | 8.2±0.6 | 3.3±1.0 | | 4.1±0.8 |  |  |
| 10 | 2.9±0.6 | 1.4±0.5 | 8.4±0.8^‡^ | 6.8±0.6^‡^ | 5.0±1.1 | | 3.0±0.8 |  |  |
| 20 | 1.3±0.7* | 1.6±0.6* | 5.3±0.9^‡^ | 5.7±0.7^‡^ | 3.2±1.2 | | 2.8±1.0 |  |  |

*main effect of treatment, *F*_2,104_=4.0, *P*<0.05; posthoc Sal vs. SCH20, *P*<0.05

^‡^main effect of treatment, *F*_2,104_=5.1, *P*<0.01; posthoc Sal vs. SCH20 and SCH10 vs. SCH20, *Ps*<0.05

**Supplemental table 3: Effect of SCH on alcohol-seeking behavior in rats trained to self-administer alcohol on FR5 and VI30 schedules.** In this study, rats were trained to self-administer alcohol on FR5 and VI30 schedules. We compared the effects of SCH in the two groups on multiple aspects of operant behavior: latency to the first lever press (s), number of active lever responses during the session, and number of EtOH deliveries earned (mean ± SEM). Behaviors were measured during operant sessions after i.p. injections of either saline (0), 10 (SCH10) or 20µg/kg SCH (SCH20) and analyzed with repeated-measures, 2-way ANOVA. Only main effects of treatment on latency and active responding were observed. Latency was reduced by either SCH10 or SCH20, while active responding was dose-dependently reduced. There was a significant interaction of SCH treatment and training schedule on EtOH deliveries earned. Specifically, VI30 rats earned more reinforcements than FR5 rats under SCH10, producing a dose-dependent effect of SCH in the VI30 group, while both doses of SCH reduced reinforcements similarly in the FR5 group.

| Training group | SCH, µg/kg | Latency (s) | Active lever responses | EtOH deliveries |
| --- | --- | --- | --- | --- |
| FR5 N=11 | 0 | 62.8 ± 50.2 | 104 ± 10^c^ | 21 ± 2 |
|  | 10 | 388.8 ± 207.6^b^ | 22 ± 8^c^ | 4 ± 1^d,e^ |
|  | 20^a^ | 221.9 ± 107.9^b^ | 12 ± 5^c^ | 2 ± 1^d,e^ |
| VI30 N=15 | 0 | 18.7 ± 8 | 132 ± 24^c^ | 23 ± 1^f^ |
|  | 10 | 239.5 ± 103.2^b^ | 37 ± 7^c^ | 13 ± 2^d,f^ |
|  | 20 | 796.7 ± 214.1^b^ | 17 ± 5^c^ | 7 ± 2^d,f^ |

^a^ N=10

^b^ P<0.001 main effect of treatment, F_2,47_=1.43; posthoc Ps<0.01 vs. Sal

^c^ P<0.001 main effect of treatment, F_2,47_=1.93; posthoc Ps<0.01 between all doses

^d^ Training schedule x treatment interaction, F_2,47_=5.9, P<0.005; posthoc Ps<0.05 between reinforcement schedules after SCH10 and SCH20

^e^ Training schedule x treatment interaction ^d^ ; posthoc Ps<0.001 vs.saline within group

^f^ Training schedule x treatment interaction ^d^; posthoc Ps<0.001 between all doses within group
